# Design, Synthesis, and Biological Evaluation of New Azulene-Containing Chalcones

**DOI:** 10.3390/ma15051629

**Published:** 2022-02-22

**Authors:** Daniela Bala, Luiza-Izabela Jinga, Marcela Popa, Anamaria Hanganu, Mariana Voicescu, Coralia Bleotu, Laszlo Tarko, Simona Nica

**Affiliations:** 1 Faculty of Chemistry, Department of Physical-Chemistry, University of Bucharest, 4-12 Bvd. Regina Elisabeta, 030018 Bucharest, Romania; dbala@gw-chimie.math.unibuc.ro; 2 “C. D. Nenitzescu” Institute of Organic Chemistry, Romanian Academy, 202B Spl. Independentei, 060023 Bucharest, Romania; jingaizabela@yahoo.com (L.-I.J.); anamaria_hanganu@yahoo.com (A.H.); tarko_laszlo@yahoo.com (L.T.); 3 Research Institute of the University of Bucharest (ICUB), 36-46 Bvd. M. Kogalniceanu, 50107 Bucharest, Romania; marcela.popa@bio.unibuc.ro (M.P.); cbleotu@yahoo.com (C.B.); 4 Faculty of Biology, Department of Botany and Microbiology, University of Bucharest, 1-3 Aleea Portocalelor, 060101 Bucharest, Romania; 5 Faculty of Chemistry, Department of Organic Chemistry, Biochemistry and Catalysis, Research Centre of Applied Organic Chemistry, University of Bucharest, 90-92 Panduri Street, 050663 Bucharest, Romania; 6 Institute of Physical Chemistry “Ilie Murgulescu” of the Romanian Academy, Splaiul Independentei 202, 060021 Bucharest, Romania; voicescu@icf.ro; 7 Stefan S. Nicolau Institute of Virology, 285 Mihai Bravu Avenue, 030317 Bucharest, Romania

**Keywords:** azulene-chalcones, synthesis, optical properties, fluorescence, antibacterial and antifungal activity

## Abstract

Azulene-containing chalcones have been synthesized via Claisen–Schmidt condensation reaction. Their chemical structure has been established by spectroscopic methods where the ^1^H-NMR spectra suggested that the title chalcones were geometrically pure and configured *trans* (J = 15 Hz). The influence of functional groups from azulene-containing chalcones on the biological activity of the 2-propen-1-one unit was investigated for the first time. This study presents optical and fluorescent investigations, QSAR studies, and biological activity of 10 novel compounds. These chalcones were evaluated for their antimicrobial activity against Gram-positive and Gram-negative bacteria. The results revealed that most of the synthesized compounds showed inhibition against Gram-negative microorganisms, independent of the substitution of azulene scaffold. Instead, all azulene-containing chalcones exhibited good antifungal activity against *Candida parapsilosis*, with MIC values ranging between 0.156 and 0.312 mg/mL. The most active compound was chalcone containing azulene moieties on both sides of the 2-propene-1-one bond, exhibiting good activity against both bacteria-type strains and good antifungal activity. This antifungal activity combined with low toxicity makes azulene-containing chalcones a new class of bioorganic compounds.

## 1. Introduction

**Scheme 1:**
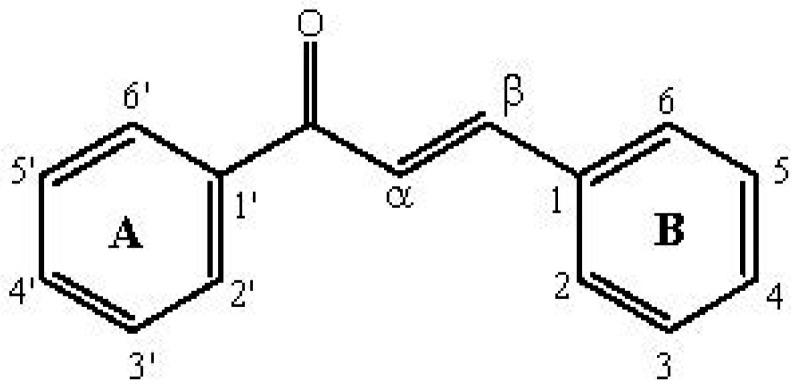
The basic chemical structure of chalcones.

The spread of antibiotic-resistant pathogenic microorganisms represents a serious concern of the scientific community; therefore, the development of newer drugs with increased effect against broad strains of bacteria is imperative. Special attention has been paid to chalcones, a class of organic compounds with a broad range of biological activities, including antibacterial, antitumor, antimalarial, anti-inflammatory, and anti-HIV activities [1,2,3,4]. Moreover, chalcones are versatile precursors in the synthesis of numerous pharmacologically active heterocycle compounds, flavonoids, and isoflavonoids [5,6]. The chemical structure consisting of two aromatic rings joined by an α,β-unsaturated carbonyl system (Scheme 1), which is available via easy organic synthesis, has generated intensive research. The biological properties of chalcones were found to be dependent on the presence, number, and position of functional groups in both A and B rings [7,8,9]. Structure–activity relationship (QSAR) studies have shown that certain biological activities can be enhanced by appending different hydrophilic or hydrophobic substituents [10,11,12]. Thus, the synthesis of tailored chalcone derivatives with appropriate biologically active moieties can provide a deeper insight into structure–activity relationships, enabling the design of effective antimicrobial compounds.

In contrast to common benzene and naphthalene aromatic units, azulene is a non-benzenoid aromatic system with unusual properties and beautiful blue color owing to intrinsic bipolar structure [13]. Azulene derivatives have attracted the interest of many research groups due to their applications in molecular switchers [12], organic transistors (FETs) [14], light-emitting diodes (LEDs) [15], supramolecular materials [16,17], or optical data storage devices [18,19]. Although azulenes are known for their biological activity in natural plant extracts [20], dopamine receptors [21], or inhibition of histamine release from mast-cell-like cells in the stomach [22], this scaffold has never been used as a pharmacophoric element in the design of biologically active chalcones.

As part of our ongoing interest in developing methodologies for isolation of azulenyl ethenes for various purposes [23,24,25,26], we have recently reported on the synthesis of 1-azulenyl-2′-azachalcones as precursors for the preparation of fluorescent 4′-azulenyl substituted terpyridines [27]. Therefore, we proposed taking advantage of the reactivity of the azulene-1-carbaldehyde and extending our studies towards the synthesis of biologically active compounds.

The aim of the present study was the synthesis of chalcones containing azulene moieties and to investigate for the first time the influence of this functional group in the biological activity of the 2-propen-1-one unit. In this context, we describe herein the chemical synthesis, optical and fluorescence investigations, QSAR studies, and biological activity of 10 novel azulene-containing chalcones.

## 2. Materials and Methods

### 2.1. Organic Synthesis

#### General Experimental Information

Unless otherwise stated, the commercially available chemicals and solvents were used as received. The azulen-carboxaldehyde derivatives were prepared according to reported procedures [13]. The IR spectra (KBr pellets) were collected on a Bruker Tensor 37 spectrometer (Ettlingen, Germany) in the 400–4000 cm^−1^ range. The NMR spectra were recorded on a Bruker Ultrashield Advance III 500 MHz spectrometer (Karlsruhe, Germany); the chemical shifts expressed as δ (ppm) values are reported relative to the residual proton NMR resonances of the deuterated solvent. Multiplicities of proton resonance are designated as singlet (s), doublet (d), triplet (t), and multiplet (m). COSY and HETCOR correlation experiments were used for the structure assignment. The absorption spectra were recorded on a Varian Cary 100 spectrophotometer (Palo Alto, CA, USA). The fluorescence emission spectra were recorded at room temperature with a Jasco FP-6500 spectrofluorometer (Mary’s Court Easton, MD, USA) equipped with a 150 W xenon lamp using 5 nm bandpasses for the excitation and the emission monochromators, detector response of 1 s, scan rate of 100 nm/min, and data pitch of 1 nm. The excitation wavelength was 425 nm. Elemental analyses were performed with Perkin Elmer CHN 240B analyzer. Mass spectra were recorded with a Varian 1200L Quadrupole MS/MS spectrometer (Palo Alto, CA, USA) by direct injection in ESI, positive mode. Melting points were determined using a Koehler Automatic Melting Point Range apparatus (New York, NY, USA), (K90190) and are uncorrected. Thin-layer chromatography (TLC) was performed on silica gel F_254_ plates (Merck, Darmstadt, Germany) with visualization by UV irradiation at 254 and 365 nm. 

##### General Procedure for the Synthesis of Chalcones

To a stirred solution of the appropriate 1-azulenecarbaldehyde (10 mmol) in ethanol (10 mL) after the complete dissolution, the appropriate aromatic methyl ketone (10 mmol) and solid KOH (10 mmol) were added. The reaction mixture was then stirred at room temperature overnight. After completion (as been monitored by TLC), the reaction mixture was filtered and washed with cold ethanol. The filtrate, containing besides unreacted raw materials additional chalcone product, was evaporated to dryness, and the crude product was purified by silica gel column chromatography with petroleum ether and ethyl acetate (3:1 *v*/*v*) as solvent system. The yields of the condensation reaction for each chalcone are listed in Tables 1 and 2, whereas relevant spectroscopic data are given in Tables 3 and 4.

*(E)-1-(2-Hydroxyphenyl)-3-phenylprop-2-en-1-one* (**1**). Synthesized according to the general procedure, using acetophenone and azulene-1-carbaldehyde. Yield: 67%, green solid, *m.p.* 160.9—162.8 °C. ^1^H-NMR (500.13 MHz, CDCl_3_, δ ppm, J Hz): 8.69 (d, 1H, H-8, 9.9 Hz), 8.54 (d, 1H, H-β, J = 15.2 Hz,), 8.35–8.33 (m, 2H, H-2 and H-4), 8.09 (d, 2H, H-2′and H-6′, 7.3 Hz), 7.70 (t, 1H, H-6, 9.8 Hz), 7.61 (d, 1H, H-α, 15.2 Hz), 7.58 (t, 1H, H-4′, 7.3 Hz), 7.52 (t, 2H, H-3′ and H-5′, 7.3 Hz), 7.46 (d, 1H, H-3, 4.3 Hz), 7.37 (t, 1H, H-7, 9.8 Hz), 7.31 (t, 1H, H-5, 9.7 Hz). ^13^C-NMR (125.77 MHz, CDCl_3_, δ ppm): 190.4 (C=O), 144.9 (C_q_), 139.8 (C_q_), 139.2( C_q_), 139.1 (C-6), 137.7 (C-2 or C-4), 136.3 (C-β), 134.7 (C-2 or C-4), 134.3 (C-8), 132.2 (C-4′), 128.5 (C-3′ and C-5′), 128.3 (C-2′ and C-6′), 126.2 (C-5), 125.5 (C-7), 125.2 (C_q_), 120.2 (C-3), 118.2 (C-α). MS-ESI positive mode: 259 (M^+^) (100%), 105 (25%), 152 (46%), 181 (30%) Selected IR data: 3056, 1643 (C=O), 1595 (C=C), 1562, 1398, 1273, 1217, 1182, 1022, 978 (C=C), 780, 692, 640 cm^−1^.

*(E)-3-(Azulen-1-yl)-1-(4-hydroxyphenyl)prop-2-en-1-one* (**2**). Synthesized following the general procedure using p-hydroxyacetophenone and azulen-1-carbaldehyde. Yield 62%, light brown solid, *m.p.* 218.7–227.9 °C. ^1^H-NMR (500.13 MHz, DMSO-d_6_, δ ppm, J Hz): 10.31 (s, 1H, -OH), 8.80 (d, 1H, H-8, 9.9 Hz), 8.65 (d, 1H, H-2, 4.2 Hz), 8.48 (d, 1H, H-4, 9.3. Hz), 8.37 (d, 1H, H-β, 15.0 Hz), 8.09 (d, 2H, H-2′ and H-6′, 8.3 Hz), 7.90 (d, 1H, H-α, 15.0 Hz), 7.82 (t, 1H, H-6, 9.9 Hz), 7.55 (d, 1H, H-3, 4.2 Hz), 7.47–7.37 (m, 2H, H-5 and H-7), 6.91 (d, 2H, H-3′ and H-5′, 8.3 Hz). ^13^C-NMR (125.77 MHz, DMSO-d_6_, δ ppm): 186.8 (C=O), 161.7 (C_q_), 144.2 (C_q_), 139.6 (C-6), 138.8 (C_q_), 137.9 (C-4), 135.5 C-2), 134.5, (C-8), 134.4 (C-β), 130.8 (C-2′ and C-8′), 129.8 (C_q_), 126.3 (C-5 or C-7), 125.7 (C-5 or C-7), 124.9 (C_q_), 120.1 (C-3), 118.2 (C-α), 115.2 (C-3′ and C-5′). MS-ESI positive mode: 275 (M^+^) (100%), 120.9 (20%). Selected IR data: 3490 (OH), 3016, 2950, 1587 (C=O), 1545 (C=C), 1515, 1402, 1281, 1167.98, 972 (C=C), 824, 774, 705 cm^−1^.

*(E)-1-(4-Aminophenyl)-3-(azulen-1-yl)prop-2-en-1-one* (**3**). Synthesized from p-aminoacetophenone and azulen-1-carbaldehyde. Yield 73%, silver solid, *m.p.* 219.7–222.9 °C. ^1^H-NMR (500.13 MHz, CDCl_3_, δ ppm, J Hz): 8.79 (d, 1H, H-8, 9.9 Hz), 8.50 (d, 1H, H-β, 15.1 Hz), 8.34 (d, 1H, H-2, 4.3 Hz), 8.32 (d, 1H, H-4, 9.4 Hz), 8.00 (d, 2H, H-2′, H-6′, 8.5 Hz), 7.67 (t, 1H, H-6, 9.8 Hz), 7.63 (d, 1H, H-α, 15.1 Hz), 7.45 (d, 1H, H-3, 4.2 Hz), 7.33 (t, 1H, H-7, 9.8 Hz), 7.29–7.26 (m, 1H, H-5, signal overlapped with CHCl_3_), 6.73 (d, 2H, H-3′, H-5′, 8.5 Hz). ^13^C-NMR (125.77 MHz, CDCl_3_, δ ppm): 188.2 (C=O), 150.6 (C_q_), 144.6 (C_q_), 139.3 (C_q_), 139.0 (C-6), 137.5 (C-4), 134.8 (C-2), 134.6 (C-β), 134.4 (C-8), 130.8 (C-2′, C-6′), 129.5 (C_q_), 125.8 (C-5), 125.6 (C_q_), 125.1 (C-7), 120.0 (C-3), 118.4 (C-α), 114.0 (C-3′, C-5′). MS-ESI positive mode: 274 (M^+^) (100%), 181 (60%), 153 (40%), 120 (30%). Selected IR data: 3439 (NH_2_), 3340, 3229, 1628, 1593 (C=C), 1545, 1438, 1273, 1176, 964 (C=C), 827, 782, 734 cm^−1^.

*(E)-3-(Azulen-1-yl)-1-(naphthalen-1-yl)prop-2-en-1-one* (**4**). Synthesized according to the general procedure using 1-acetylnaphthalene and azulen-1-carbaldehyde. Yield 75%, brown oil, *m.p.* 89.9–98.9 °C. ^1^H-NMR (500.13 MHz, CDCl_3_, δ ppm, J Hz): 8.50 (d, 1H, H-8, 9.9 Hz), 8.38 (d, 1H, H-8′, 9.2 Hz), 8.33–8.29 (m, 3H, H-2, H-4, H-β), 8.00 (d, 1H, H-2′, 8.2 Hz), 7.93 (d, 1H, H-5′, 9.3 Hz), 7.83 (d, 1H, H-4′, 7.0 Hz), 7.68 (t, 1H, H-6, 9.9 Hz), 7.58–7.54 (m, 3H, H-3′, H-6′, H-7′), 7.44 (d, 1H, H-3, 4.3 Hz), 7.37 (d, 1H, H-α, 15.4 Hz), 7.32–7.28 (m, 2H, H-5, H-7, 9.8 Hz). ^13^C-NMR (125.77 MHz, CDCl_3_, δ ppm): 195.8 (C=O), 145.0 (C_q_), 139.7 (C_q_), 139.1 (C-6), 138.4 (C_q_), 137.7 (C-2, C-4 or C- β), 137.3 (C-2, C-4 or C- β), 135.0 (C-2, C-4 or C- β), 134.1 (C-8), 133.9 (C_q_), 130.9 (C-2′), 130.6 (C_q_), 128.4 (C-5′), 127.1 (C-3′, C-6′ or C-7′), 126.6 (C-5 or C-7), 126.3 (C-4′), 125.9 (C-3′, C-6′ or C-7′), 125.6 (C-8′), 124.8 (C-5 or C-7), 124.7 (C-3′, C-6′ or C-7′), 123.6 (C-α), 120.4 (C-3). MS-ESI positive mode: 309 M^+^ (100%), 181 (48%), 155 (44%), 308 (24%). Selected IR data: 3434, 3045, 2924, 1678 (C=O), 1557 (C=C), 1397, 1280, 1175, 965 (C=C), 864, 804, 744 cm^−1^.

*(E)-3-(Azulen-1-yl)-1-(naphthalen-2-yl)prop-2-en-1-one* (**5**). Synthesized using 2-acetylnaphthalene and azulen-1-carbaldehyde. Yield 99.99%, brown solid, m.p. 183.9–193.7 °C. ^1^H-NMR (500.13 MHz, CDCl_3_, δ ppm, J Hz): 8.72 (d, 1H, H-8, 9.9 Hz), 8.61–8.59 (m, 2H, H-β, H-1′), 8.43 (d, 1H, H-2, 4.3 Hz), 8.36 (d, 1H, H-4, 9.4 Hz), 8.17 (d, 1H, H-3′, 8.8 Hz), 8.03 (d, 1H, H-8′, 7.9 Hz), 7.97 (d, 1H, H-4′, 8.6 Hz), 7.91 (d, 1H, H-5′, 8.0 Hz), 7.78 (d, 1H, H-α, 15.1 Hz), 7.71 (t, 1H, H-6, 9.9 Hz), 7.62–7.56 (m, 2H, H-6′ and H-7′), 7.48 (d, 1H, H-3, 4.3 Hz), 7.38 (t, 1H, H-7, 9.9 Hz), 7.31 (t, 1H, H-5, 9.8 Hz). ^13^C-NMR (125.77 MHz, CDCl_3_, δ ppm): 190.2 (C=O), 145.0 (C-10), 139.9 (C-9), 139.1 (C-6), 137.7 (C-4), 136.5 (C-10′), 136.3 (C-β), 135.30 (C-9′), 134.8 (C-2), 134.4 (C-8), 132.7 (C-2′), 129.5 (C-8′), 129.4 (C-1′), 128.4 (C-4′), 128.0 (C-6′ or C-7′), 127.8 (C-5′), 126.6 (C-6′ or C-7′), 126.2 (C-5), 125.6 (C-7), 125.3 (C-1), 124.7 (C-3′), 120.3 (C-3), 118.3 (C-α). MS-ESI positive mode: 309 (M^+^) (100%), 181 (50%), 155 (30%). Selected IR data: 3054, 1642 (C=O), 1562 (C=C), 1398, 1216, 1055, 975 (C=C), 898, 759, 742 cm^−1^.

*(E)-1,3-Di(azulen-1-yl)prop-2-en-1-one* (**6**). Similarly to Type I chalcones, equimolar amounts of 1-acetylazulene and azulen-1-carbaldehyde were reacted under the same conditions. Yield 84%, black solid, *m.p.* 185.4 °C. ^1^H-NMR (500.13 MHz, CDCl_3_, δ ppm, J Hz): 10.08 (d, 1H, H-8′, 9.9 Hz), 8.74 (d, 1H, H-8, 9.9 Hz), 8.56–8.49 (m, 3H, H-2′, H-4′ and H-β), 8.40 (d, 1H, H-2, 4.2 Hz), 8.32 (d, 1H, H-4, 9.3 Hz), 7.85–7.82 (m, 2H, H-6′ or H-α), 7.67 (t, 1H, H-7′, 9.9 Hz), 7.64 (t, 1H, H-6, 9.9 Hz), 7.50–7.46 (m, 2H, H-3′ or H-5′), 7.37 (d, 1H, H-3, 4.1 Hz), 7.34 (t, 1H, H-7, 9.9 Hz), 7.29–7.26 (m, 1H, H-5, overlapped with CHCl_3_ signal). ^13^C-NMR (125.77 MHz, CDCl_3_, δ ppm): 186.8 (C=O), 145.3 (C_q_), 144.5 (C_q_), 140.9 (C_q_), 139.9 (C-2′, C-4′ or C- β), 139.5 (C-8′), 139.48 (C-6′), 139.1 (C_q_), 138.9 (C-6 or C-7′), 138.4 (C-2′, C-4′ or C- β), 137.4 (C-4), 134.6 (C-2), 134.2 (C-8), 133.3 (C-2′, C-4′ or C- β), 129.0 (C-6 or C-7′), 127.2 (C-3′ or C-5′), 127.1 (C_q_), 125.8 (C_q_), 125.6 (C-5), 125.0 (C-7), 122.1 (C-α), 119.9 (C-3′ or C-5′), 117.9 (C-3). MS-ESI positive mode: 309 (M^+^) (100%), 181 (75%), 157 (46%), 108 (30%). Selected IR data: 2856, 1620 (C=O), 1549, 1396, 1283, 976 (C=C) cm^−1^.

*(E)-1-(Azulen-1-yl)-3-(3-chloroazulen-1-yl)prop-2-en-1-one* (**7**). Synthesized similarly to 6, using 1-acetylazulene and 3-chloroazulene-1-carbaldehyde following the above described general procedure. Yield 78%, dark brown solid, *m.p.* 229.6–233.2 °C. ^1^H-NMR (500.13 MHz, CDCl_3_, δ ppm, J Hz): 10.07 (d, 1H, H-8′, 9.9 Hz), 8.68 (d, 1H, H-8, 9.9 Hz), 8.52–8.50 (m, 2H, H-2′ and H-4′), 8.64 (d, 1H, H- β, 15.1 Hz), 8.39 (d, 1H, H-4, 9.5 Hz), 8.28 (s, 1H, H-2), 7.85 (t, 1H, H-6′, 9.6 Hz), 7.82 (d, 1H, H-α, 15.3 Hz), 7.71 (t, 1H, H-6, 9.9 Hz), 7.65 (t, 1H, H-7′, 9.9 Hz), 7.50 (t, 1H, H-5′, 9.6 Hz), 7.38 (d, 1H, H-3′, 4.1 Hz), 7.33–7.28 (m, 2H, H-5 and H-7). ^13^C-NMR (125.77 MHz, CDCl_3_, δ ppm): 186.4 (C=O), 145.5 (C_q_), 141.1 (C_q_), 140.4 (C-6), 139.9 (C-8′), 139.6 (C-6′), 139.5 (C-2′ or C-4′), 138.5 (C-2′ or C-4′), 137.9 (C_q_), 137.7 (C_q_), 135.4 (C-4), 135.1 (C-8), 132.1 (C-2), 131.9 (C-β), 129.2 (C-7′), 128.4 (C-5′), 126.9 (C_q_), 125.5 (C-5 or C-7), 125.3 (C-5 or C-7), 123.5 (C_q_), 122.8 (C-α), 119.4 (C_q_), 118.0 (C-3′). MS-ESI positive mode: 343 (M^+^) (100%), 215 (75%), 128 (60%), 345 (40%). Selected IR data: 3445, 2918, 1628 (C=O), 1399 (C=C), 1028, 958 (C=C), 867, 741, 615 cm^−1^.

*(E)-1-(Azulen-1-yl)-3-(3-bromoazulen-1-yl)prop-2-en-1-one* (**8**). Synthesized following the above described general procedure using equimolar amounts of 1-acetylazulene and 3-bromoazulene-1-carbaldehyde. Yield 74%, black solid, *m.p.* 206.7–213.3 °C. ^1^H-NMR (500.13 MHz, CDCl_3_, δ ppm, J Hz): 10.07 (d, 1H, H-8’, 9.9 Hz), 8.68 (d, 1H, H-8, 9.9 Hz), 8.51–8.49 (m, 2H, H-2′ and H-4′), 8.46 (d, 1H, H-β, 15.1 Hz), 8.37–8.35 (m, 2H, H-2 and H-4), 7.85 (t, 1H, H-6′, 9.8 Hz), 7.79 (d, 1H, H-α, 15.1 Hz), 7.71 (t, 1H, H-6, 9.8 Hz), 7.65 (t, 1H, H-7′, 9.9 Hz), 7.50 (t, 1H, H-5′, 9.7 Hz), 7.37–7.33 (m, 3H, H-3′, H-5 and H-7). ^13^C-NMR (125.77 MHz, CDCl_3_, δ ppm): 186.3 (C=O), 145.5 (C_q_), 141.1 (C_q_), 140.3 (C-6), 139.9 (C-8′), 139.6 (C-2′ or C-4′), 139.56 (C_q_), 139.5 (C-6′), 138.6 (C_q_), 138.4 (C-2′ or C-4′), 137.1 (C-2 or C-4), 135.3 (C-2 or C-4), 134.8 (C-8), 131.8 (C-β), 129.2 (C-7′), 127.4 (C-5′), 126.8 (C_q_), 125.8 (C-3′, C-5 or C-7), 125.6 (C-3′, C-5 or C-7), 124.8 (C_q_), 122.8 (C-α), 118.1 (C-3′, C-5 or C-7), 107.2 (C_q_). MS-ESI positive mode: 387 (M^+^) (100%), 258.9 (80%), 389 (75%), 260.9 (50%). Selected IR data: 1626 (C=O), 1567 (C=C), 1383, 1304, 1112, 957 (C=O), 809, 739 cm^−1^.

*(E)-1-(Azulen-1-yl)-3-(3-methylazulen-1-yl)prop-2-en-1-one* (**9**). Synthesized by general condensation reaction protocol from 1-acetylazulene and 3-methylazulene-1-carbaldehyde. Yield 63%, brown solid, *m.p.* 97.2–103.9 °C. ^1^H-NMR (500.13 MHz, CDCl_3_, δ ppm, J Hz): 10.07 (d, 1H, H-8′, 9.8 Hz), 8.63 (d, 1H, H-8, 9.8 Hz), 8.53–8.49 (m, 3H, H-2′, H-4′ and H-β), 8.22 (s, 1H, H-2), 8.19 (d, 1H, H-4, 9.4 Hz), 7.83 (d, 1H, H-6′, 9.8 Hz), 7.79 (d, 1H, H-α, 15.0 Hz), 7.65–7.58 (m, 2H, H-6 and H-7′), 7.47 (t, 1H, H-5′, 9.6 Hz), 7.36 (d, 1H, H-3′, 4.1 Hz), 7.23–7.17 (m, 2H, H-5 and H-7), 2.69 (s, 3H, H-11). ^13^C-NMR (125.77 MHz, CDCl_3_, δ ppm): 186.9 (C=O), 145.2 (C_q_), 141.6 (C_q_), 140.9 (C_q_), 139.9 (C-8′), 139.5 (C_q_), 139.47 (C-6′), 139.4 (C-2′, C-4′ or C-β), 138.8 (C-6 or C-7′), 138.3 (C-2′, C-4′ or C-β), 135.1 (C-2), 134.3 (C-4), 133.8 (C-8), 133.0 (C-2′, C-4′ or C-β), 128.9 (C-6 or C-7′), 128.1 (C_q_), 127.2 (C_q_), 127.1 (C-5′), 124.3 (C-5 or C-7), 124 (C_q_), 121.7 (C-α), 117.9 (C-3′), 12.8 (C-11). MS-ESI positive mode: 323 (M^+^) (100%), 195 (75%), 154 (26%), 324 (23%). Selected IR data: 3413, 2923, 2852, 1621 (C=O), 1551 (C=C), 1396, 963 (C=C), 741 cm^−1^.

*(E)-3-(Azulen-1-yl)-1-(3-methylazulen-1-yl)prop-2-en-1-one* (**10**). Synthesized following the above described general procedure using 1-(3-methylazulen-1-yl)ethanone and azulen-1-carbaldehyde described above. Yield 57%, brown solid, *m.p.* 158.2–168.7 °C. ^1^H-NMR (500.13 MHz, CDCl_3_, δ ppm, J Hz): 9.99 (d, 1H, H-8′, 9.8 Hz), 8.71 (d, 1H, H-8, 9.9 Hz), 8.54 (d, 1H, H-β, 15.1 Hz), 8.39 (d, 1H, H-2, 4.2 Hz), 8.36–8.34 (m, 2H, H-2′ and H-4′), 8.29 (d, 1H, H-4, 9.4 Hz), 7.79 (d, 1H, H-α, 15.1 Hz), 7.75 (t, 1H, H-6′, 9.8 Hz), 7.64 (t, 1H, H-6, 9.9 Hz), 7.53 (t, 1H, H-7′, 9.8 Hz), 7.45 (d, 1H, H-3, 4.2 Hz), 7.39 (t, 1H, H-5′, 9.7 Hz), 7.30 (t, 1H, H-7, 9.8 Hz), 7.23 (t, 1H, H-5, 9.8 Hz), 2.67 (s, 3H, H-11′). ^13^C-NMR (125.77 MHz, CDCl_3_, δ ppm): 186.4 (C=O), 144.4 (C_q_), 142.0 (C_q_), 141.3 (C_q_), 140.2 (C-2′ or C-4′), 139.19 (C-8′), 139.18 (C-6′), 139.0 (C_q_), 138.8 (C-6), 137.3 (C-4), 135.2 (C-2′ or C-4′), 134.6 (C-2), 134.3 (C-8), 133.0 (C- β), 128.4 (C-7’), 125.9 (C-5′), 125.8 (C_q_), 125.7 (C_q_), 125.5 (C-5), 125.2 (C_q_), 124.8 (C-7), 122.2 (C-α), 119.9 (C-3), 12.67 (C-11′). MS-ESI positive mode: 323 (M^+^) (90%), 181 (100%), 367(30%). Selected IR data: 3065, 2919, 1616 (C=O), 1573 (C=C), 1543, 1425, 1395, 1287, 1193, 966 (C=C), 860, 743 cm^−1^.

### 2.2. Biology

#### 2.2.1. Determination of Minimum Inhibitory Concentration

The antimicrobial activity of all compounds was assessed on Gram-positive bacteria (*Staphylococcus aureus* ATCC 25923), Gram-negative bacteria (*Escherichia coli* ATCC 25922, *Pseudomonas aeruginosa* ATCC 27853), and fungi (*Candida parapsilosis* ATCC 22019) using quantitative binary serial dilution method in 96-well plates [28,29]. The negative control was represented by wells with culture medium, while positive control was the microbial strain in broth. Briefly, binary serial dilutions of the compounds performed in 100 μL of broth were seeded with microbial inoculum of 10^6^ colony forming units (CFU)/mL and incubated for 24 h at 37 °C. Minimum inhibitory concentration (MIC) values were determined as being the lowest concentration at which the tested compounds inhibited the growth of the microbial cultures compared to the positive control. The minimum inhibitory concentration values (MICs) (in µg/mL) for 1–10 are summarized in Table 5. Due to the fact that the obtained compounds were colored, after the incubation of the 96-well plates, from each well, 10 µL were seeded on agar plates (Plate Count Agar) to determine the inhibitory concentration. The same value of the inhibitory concentration was obtained in repeated experiments.

#### 2.2.2. Determination of Antibiofilm Activity

The effect of the chalcones on the biofilm formation was evaluated after the determination of the MICs. Briefly, the plates were emptied, washed to remove the nonadherent cells, fixed with methanol, and stained with crystal violet. The antibiofilm activity was determined after adding 33% acetic acid solution by measuring the absorbance at 490 nm [30], and the results are listed in Table 6.

#### 2.2.3. Cytotoxicity Evaluation

##### Cell Viability Assay

To assess the toxicity of the azulene-containing chalcones, the adherent cell line HEp-2 (American Cell Type Collection [ATCC] CCL-23^TM^) was used. Adherent cells were trypsinized, counted, and seeded in 96-well plates (NUNC, USA) at a density of 1 × 10^4^ cells per well in Dulbecco’s modified medium (DMEM:F12) (Sigma, St. Louis, MO, USA) supplemented with 10% thermally inactivated fetal bovine serum (FBS) ((Sigma, St. Louis, MO, USA) and chalcone at concentrations of 200, 100, 50, and 25 μg/mL. After cells were maintained for 48 h at 37 °C in humidified 5% CO_2_, cell viability was evaluated using the CellTiter Glo kit (Promega, Madison, WI, USA), according to the manufacturer’s indications. The 50% cytotoxic concentration (IC50) was determined from the dose–response curve.

##### Cell Cycle Analysis

For cell cycle analysis, a suspension of 7.5 × 10^4^ HEp-2 cells was seeded for 24 h in a 1 cm^2^ well in DMEM:F12 containing 10% fetal bovine serum. After recovery, the cells were treated with 50 µg/mL or 100 µg/mL azulene-containing chalcone, and 24 h later, the cells were harvested, fixed in cold ethanol overnight, and stained with RNase A/propidium iodide at a concentration of 1 mg/mL to 100 µg/mL. The events were recorded using the Beckman Coulter flow cytometer and analyzed using FlowJo 7.2.5 software.

##### Mitotracker—Hoechst Stain

The HEp-2 cells were seeded at a concentration of 7.5 × 10^4^ in DMEM:F12 containing 10% FBS and treated with azulene-containing chalcone as for cell cycle analysis. After 24 h, the cells were stained with 20 nM MitoTracker Red CMXRos (Sigma, St. Louis, MO, USA) and 10 µg/mL Hoechst 33342 (Sigma, St. Louis, MO, USA), 20 min, at 37 °C. The evaluation was conducted using an Observer D1 Zeiss microscope with a fluorescence module.

##### Gene Expression Quantification

The mechanism of azulene-containing chalcone toxicity was assessed by gene expression evaluation. HEp-2 cells were seeded in 6-well plates at a concentration of 3 × 10^5^ cells/wells in DMEM:F12 supplemented with 10% FBS. The cells were treated with azulene-containing chalcone at a concentration of 100 µg/mL. After 24 h, the cells were harvested and centrifuged, and the pellet was subjected to total RNA extraction using Trizol reagent (Sigma, St. Louis, MO, USA). The cDNA was produced using 2 µg total RNA and High Capacity Reverse Transcription Kit (Thermo Fisher Scientific, Chelmsford, MA, USA). Fifty nanograms of cDNA was subjected to PCR reaction using Maxima SYBR Green/ROX qPCR Master Mix (Thermo Fisher Scientific, Chelmsford, MA, USA) and Origene primer pairs: caspase 3 (CAT#: HP207674) caspase 7 (CAT#: HP233752), caspase 8 (CAT#: HP234494), caspase 9 (CAT#: HP205155), caspase 10 (CAT#: HP228052). For NOX-4 and NOS2, the following primers were used: NOX 4 forward 5′-TATCACTACCTCCACCAGATGT-3 and reverse 5′-AGGCTGCAGTTGAGGTTAAGAA-3′ and NOS2 forward 5′-TGAACTACGTCCTGTCCCCT-3′ and reverse 5′-CTCTTCTCTTGGGTCTCCGC-3′. Amplification was completed using StepOne Real-Time PCR System (Thermo Fisher Scientific, Chelmsford, MA, USA). The relative changes in the levels of mRNA expression were quantified by using the 2^−ΔΔCT^ method.

### 2.3. Quantitative Structure–Activity Relationship (QSAR) Studies

The QSAR studies were carried out on 48 chalcones, including 10 synthesized azulenyl-chalcones, in order to identify the physicochemical parameter responsible for their antimicrobial activity against *Staphylococcus aureus* [31,32,33,34,35]. The geometry optimization of the molecules was completed using PCModel and MOPAC programs [36,37]. The descriptors were calculated using MOPAC, PRECLAV, and DRAGON software, and the descriptors and QSAR equations were selected using the PRECLAV algorithm. The best QSAR model was chosen based on the statistical parameters such as the square of the correlation coefficient (r^2^), the Fischer’s value of significance (F), and the standard error of estimate(s).

## 3. Results and Discussion

**Scheme 2:**
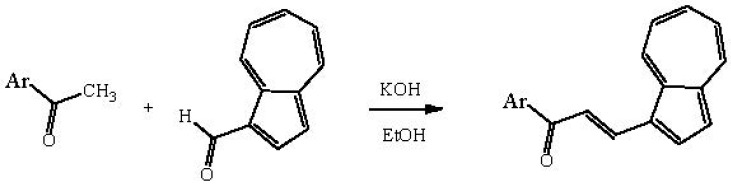
Synthesis of Type I azulene-containing chalcones.

**Scheme 3:**
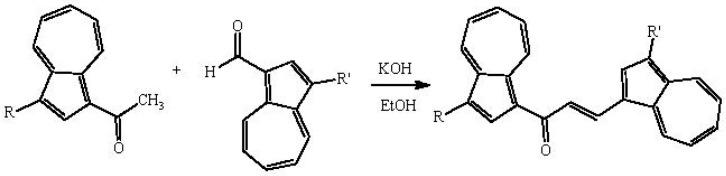
Synthesis of Type II azulene-containing chalcones.

The most convenient method to prepare chalcones is the Claisen–Schmidt condensation of equimolar amounts of aromatic methyl ketones with substituted aldehydes in an alkali ethanol solution, at room temperature [38]. Following this synthetic strategy, we synthesized 10 azulene-containing chalcones divided into Type I and Type II compounds (Scheme 2 and Scheme 3) by replacing one or both rings of the 1,3-diphenyl-2-propen-1-one parent compound (see Scheme 1) with azulene groups. For the first time, the azulene-containing chalcones were evaluated for their biological properties, namely their antibacterial properties using four different cultures. To our knowledge no QSAR study involving azulene-containing chalcone derivatives has been reported so far; therefore, we report on our efforts to generate quantitative structure–activity relationship information on the antibacterial effect of these chalcones against *Staphylococcus aureus*.

### 3.1. Chemistry

We initially explored the reaction of azulene-1-carboxaldehyde with various acetophenones in the presence of stoichiometric amounts of potassium hydroxide, obtaining Type I chalcones (Scheme 2 and Table 1). The yields of the reaction are between 62 and 73% (entries 1–3), increasing for the 4-hydroxy substitution of the phenyl group. In order to study the effect of different aromatic substitutions, we also prepared chalcones **4** and **5** that contain 1- and 2-naphthyl moieties, respectively, for a direct comparison with the phenyl analog.

Next, we synthesized 1,3-diazulenyl-2-propen-1-one (Type II) compounds, wherein both rings A and B are azulenyl groups (Scheme 3). These chalcones were prepared through condensation of 1-acetylazulene derivatives (indicated as R) with substituted azulen-1-carboxaldehyde (indicated as R′) in ethanolic potassium hydroxide solution. By comparison with the corresponding phenyl-containing chalcone, condensation of 1-acetylazulene afforded the corresponding chalcone **6** in higher yield (Table 2). Instead, substitution of the azulene in the starting materials caused a decrease in the condensation reaction yield, the most significant effect being observed for 3-methyl-1-acetylazulene, when the corresponding chalcone **10** was isolated in 57% yield.

The molecular structure of all the isolated azulene-chalcone compounds was confirmed by IR, NMR, and MS spectroscopy. The characteristic stretching vibration band of the chalcone moiety [39] was observed in all cases as a prominent band between 1625 and 1650 cm^−1^ in the IR spectrum of each. A strong stretching band attributed to the C=C chalcone bond appeared at 1570–1600 cm^−1^. For chalcones **2** and **3**, a broad signal was observed around 3400 cm^−1^ due to the presence of OH or NH_2_ group, respectively. Mass spectra were recorded using electrospray ionization and showed the correct molecular ion peaks in all cases.

All synthesized compounds were fully characterized by ^1^H- and ^13^C-NMR spectroscopy. Assignment of the peaks was accomplished by COSY, HMQC, and HMBC experiments. In all cases, the characteristic signals of the vinylic protons H-α and H-β with coupling constants around 15 Hz were observed in the ^1^H-NMR spectra, indicating an *E* configuration. Their δ values are influenced by the chalcone A ring, a deshielding effect being observed from phenyl to 2-naphthyl to 1-azulenyl. An exception was observed in the case of the 1-naphthyl group when both H-α and H-β vinylic protons were shielded when compared to the other aromatic A ring of chalcones. Instead, substitution of the azulenyl rings, either A or B rings with electronegative halogens (chalcones **7** and **8**) or with an electron-donating group (chalcones **9** and **10**), affects the chemical shifts of the vinylic protons to a small extent. As expected, the aromatic protons of the azulenyl B ring are also affected by the conjugation as well as the electronic effects of the substituents present on the ring. Table 3 summarizes the chemical shifts of the relevant protons in the herein described compounds.

The ^13^C-NMR spectra confirm the structure of the azulene-containing chalcones. The most deshielded signal is the one corresponding to the carbonyl carbon and appears in the 186.4–195.8 ppm range, the expected region for δ C=O in chalcones [40]. The α- and β-carbon atoms with respect to the carbonyl group give rise to characteristic signals between 118.2 and 123.3 ppm for C-α and between 131.8 and 139.9 ppm for C-β, respectively (see experimental part).

#### Optical and Fluorescence Properties

**Figure 1:**
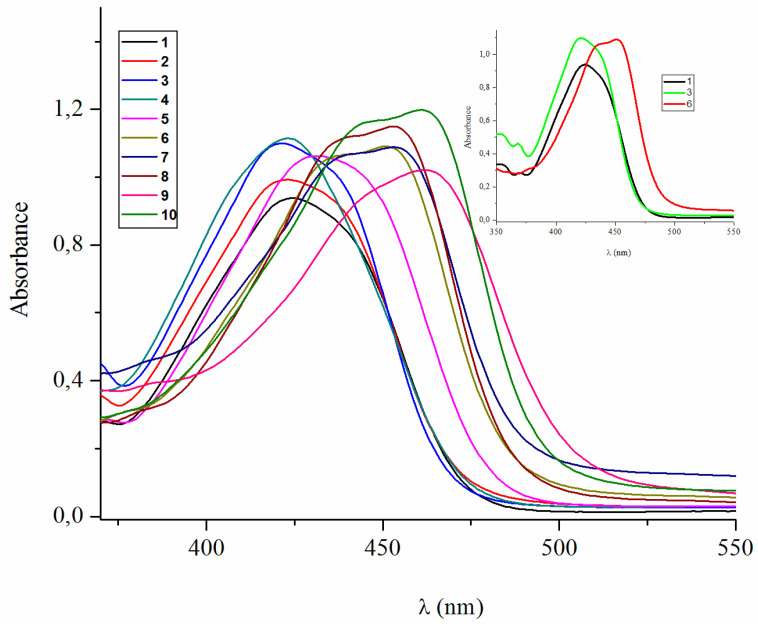
Visible absorption spectra of azulene-chalcone derivatives **1**–**10** in dichloromethane.

The absorption profiles and the emission spectra of these azulene-containing chalcones are similar and are mainly dominated by the nature of the conjugation length. In general, the UV spectrum of chalcones consists of two essential absorption bands: band I between 340 and 390 nm and a minor band II that appears between 220 and 270 nm [41]. Structural modification of the chalcone moiety results in notable changes in the π-conjugated length which results in redshifts in the absorption spectra. The visible regions of the recorded absorption spectra of chalcones **1**–**10** are shown in Figure 1, and the detailed data are listed in Table 4 (the full UV-Vis spectra are shown in Figure S1 in Supplementary Materials).

Substitution of one phenyl ring in the parent 1,3-diphenyl-2-propen-1-one chalcone with azulene moiety (chalcone **1**) produced a strong bathochromic effect of over 100 nm of the absorption band I [42]. Instead, *para*-substitution of the phenyl ring with an electron-donating group caused no shift of the longwave absorption band (chalcones **2** and **3**). The same effect is observed when replacing the phenyl ring with a 1- or 2-naphthyl group, although the system gains increased conjugation length. As expected, the substitution of both phenyl rings of the parent 1,3-diphenyl-2-propen-1-one chalcone with azulene moieties resulted in more redshift of the long-wavelength absorption band. Compared with chalcone **1**, the maximum absorption band of **6** is redshifted by 27 nm and split into two bands. For the same conjugated chalcone system, the introduction of a strong electron-donating substituent lowered the energy, which is observed as a redshift of the relevant absorption band I. For instance, the methyl substitution either on azulene ring A or azulene ring B caused a similar bathochromic effect of around 10 nm (chalcones **9** and **10**), whereas chloro- or bromo-substituents have a smaller effect on the electronic properties (Table 4).

**Figure 2:**
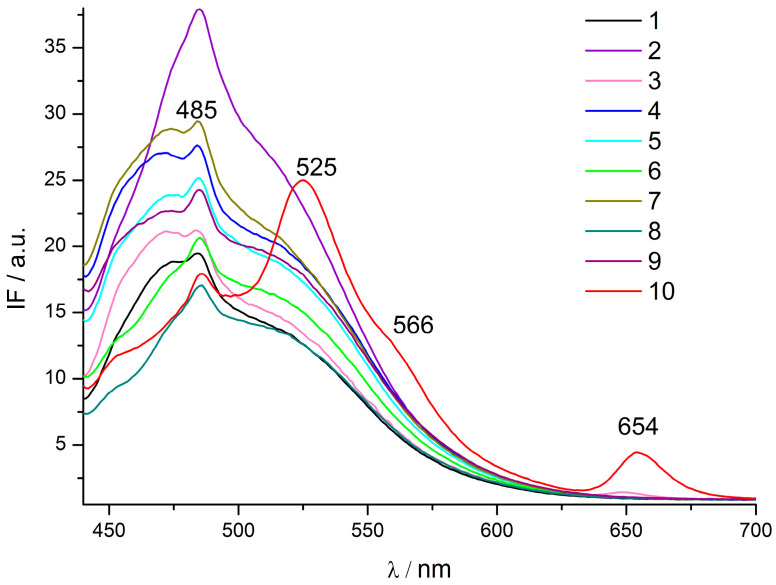
Fluorescence emission spectra of azulene-chalcone derivatives **1**–**10**.

The fluorescence spectra of the compounds were measured upon the excitation at 425 nm and are presented in Figure 2, with maximum emission bands pointed out in Table 4. It can be seen that the azulene-chalcone compounds show weak fluorescent emission maxima at 485, 525, 566, and 654 nm, more or less with the same pattern, as a function of several substitutions of the azulene fragment. Comparing **1**, **2**, and **3** chalcone derivatives, we observed that the presence of an –OH group at the benzene A ring, in the proximity of the ketone group, leads to an increase in the fluorescence emission of the azulene fragment, with the emission bands at 454, 471, 485, and 525 nm. A change of the substitution with the NH_2_ group leads to weak fluorescence emission at ~650 nm. No major changes in the fluorescence emission were observed for 1- or 2-naphthyl substitution of the azulene-chalcone moiety. Compound **9** containing two azulene rings exhibits stronger fluorescence emission when compared with chalcone **1**, but with the same fluorescence profile. In direct comparison, the fluorescence emissions of **7**, **8**, and **10**, where the substituents are placed on the azulene ring B, exhibit a similar fluorescence emission pattern with maxima at 485 and 525 nm, with no major changes produced by the substituent (-CH_3_, Cl, Br). However, the bromine caused a pronounced fluorescence quenching of the azulene fragment, whereas for chalcones **7** and **10** an iso-emissive point at ~557 nm was evidenced. It is worth observing that the position of the –CH_3_ group on the azulene ring A leads to an increase in the fluorescence emission at 525 nm, with the appearance of new emission bands at 566 and 654 nm.

Overall, the studied chalcone derivatives showed fluorescent properties in the 450–700 nm range, depending on the number of azulene fragments and the substituent position in the azulene fragment and position relative to the ketone group. The studied chalcone derivatives exhibited different π-electron delocalization abilities, in which a better π-electron delocalization in the proximity of the ketone group contributes to better fluorescence emission.

### 3.2. Biological Evaluation

#### 3.2.1. Antimicrobial Activity

The antibacterial effect of the azulene moiety introduced in the chalcone structure was investigated against Gram-positive (*Staphylococcus aureus* ATCC 25923) and Gram-negative (*Escherichia coli* ATCC 25922, *Pseudomonas aeruginosa* ATCC 27853) bacteria, while the antifungal effect was tested against *Candida parapsilosis* ATCC 22019. To our knowledge, the antibacterial activity of azulene-containing chalcone compounds has not been reported previously; moreover, this is the first investigation of the biological properties of azulene-containing chalcones in general. The results expressed in terms of minimum inhibitory concentration (MIC) were determined by in vitro screening of the antimicrobial properties using the broth microdilution method [28] and are summarized in Table 5.

The substituent in the B ring, systematically changed to obtain a set of analogs, affects the antibacterial effect of the azulene-containing chalcones to a small extent. Although it has been reported that the introduction of a hydroxyl group at the 4′ position of the benzene ring is important to the antibacterial activity [33], chalcone **2** showed not much improvement of the antimicrobial activity against all studied microorganisms. As the B ring was changed from phenyl to naphthyl, the antibacterial activity was not much improved (compounds **1**, **4**, and **5**). Compound **6**, which contains azulene moieties on both rings, and compound 7 with substitution in the azulene A ring seem more active against tested bacteria. All other investigated chalcone compounds showed similar, moderate antimicrobial activity.

We further tested the azulene-containing chalcone compounds for antifungal activity against *Candida parapsilosis* ATCC 22019. In general, the tested chalcones exhibited good antifungal activity, with MIC values ranging between 0.156 and 0.312 mg/mL. The most active compound was chalcone **6** with an MIC value of 0.156 mg/mL.

The azulene-chalcone derivatives were tested for their ability to affect biofilm formation since 80% of the world’s microbial biomass is found in the biofilm state [43]. The most efficient compound was again chalcone **6** with MBEC of 0.156–0.625 mg/mL (Table 6).

#### 3.2.2. Cytotoxicity Assay

**Figure 3:**
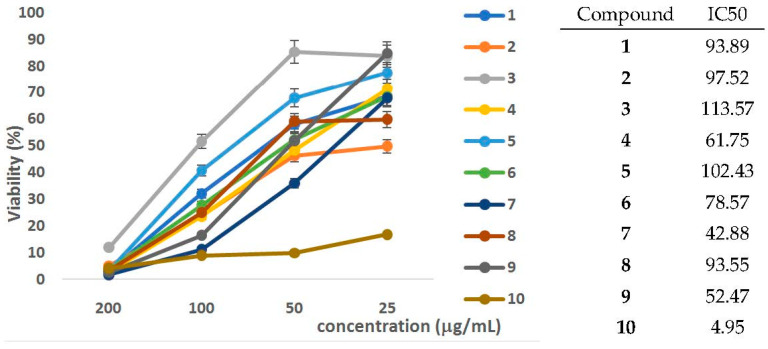
Graphical representation of cell viability percentage vs. concentration for compounds **1**–**10** (left); IC_50_ values (right).

In order to estimate the cytotoxicity of the compounds, HEp-2 cells (American Cell Type Collection (ATCC) CCL-23) were exposed to the azulene-containing chalcones at concentrations of 200, 100, 50, and 25 μg/mL and evaluated using CellTiter Glo kit (Promega, Madison, WI, USA) assay. The results presented in Figure 3 are expressed as viability percentages versus control.

The most toxic compound was compound **10**, containing substituted azulene B ring, followed by compound **7** with chloride content in the molecular structure and chalcone **9**. However, chalcone **6**, which showed efficient antibacterial and antifungal activity, revealed low toxicity with IC_50_ of 78.57 μg/mL.

**Figure 4:**
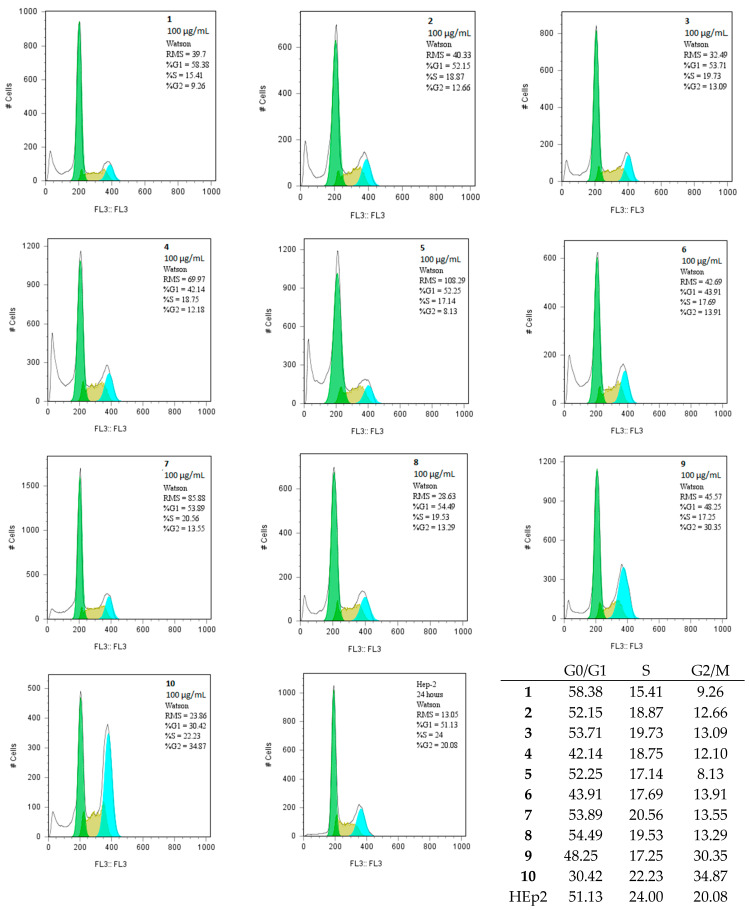
The effect of the azulene-containing chalcones **1**–**10** on the cell cycle for HEp2. Histograms show the changes in cellular DNA content after treatment with 100 µg/mL azulene-containing chalcone.

The cytotoxicity data were also confirmed by cell cycle analysis. Among the tested compounds, chalcone **10** induced an increase in the G2/M phase starting from a concentration of 50 µg/mL (Supplementary Material Figure S2), the effect increasing slightly with the concentration of the tested compound. The increase in the G2/M phase was also observed in the case of compound **9**, but at a higher concentration (100 µg/mL), but this effect was lower (Figure 4). The sub-G0 peak, on the left of G0/G1, associated with programmed cell death, was observed for all compounds when the treatment was performed at a concentration of 100 µg/mL, suggesting that a mechanism other than G2/M arrest is involved in the induction of the cellular death.

**Figure 5:**
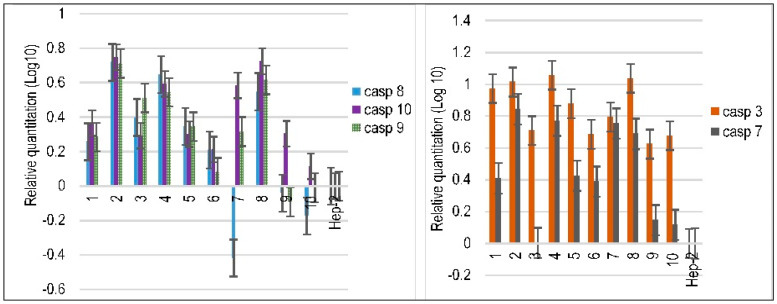
The intrinsic and extrinsic apoptotic caspase activation by 100 µg/mL azulene-containing chalcones.

We observed that the expression of both executioner caspases, caspase 3 (Casp 3), and caspase 7 (Casp 7), was activated by azulene (Figure 5, right). The production of procaspase 3 and 7 mRNA in the case of compounds **9** and **10** was quite reduced, probably correlated with the decreased number of cells. Our results show that both apoptosis-inducing pathways are activated by azulene The caspases 8 and 10 are involved in the extrinsic apoptosis pathway (Figure 5, left) *via* ligand binding to their corresponding death receptors, formation of the death-inducing signaling complex (DISC), and subsequent caspase 3 activation [44].

Production of procaspase 8 mRNA was inhibited by **7**, **9**, and **10**, with an important role in extrinsic apoptosis induction remaining for caspase 10. Cellular stress induces intrinsic apoptosis by caspase 9 activation that stimulates the caspase cascade, culminating in activation of executioner caspases 3 and 7 [42]. The mRNA expression of caspase 9 was activated following the treatment with 100 µg/mL of compounds **1**–**8** but inhibited by **9** and **10**.

**Figure 6:**
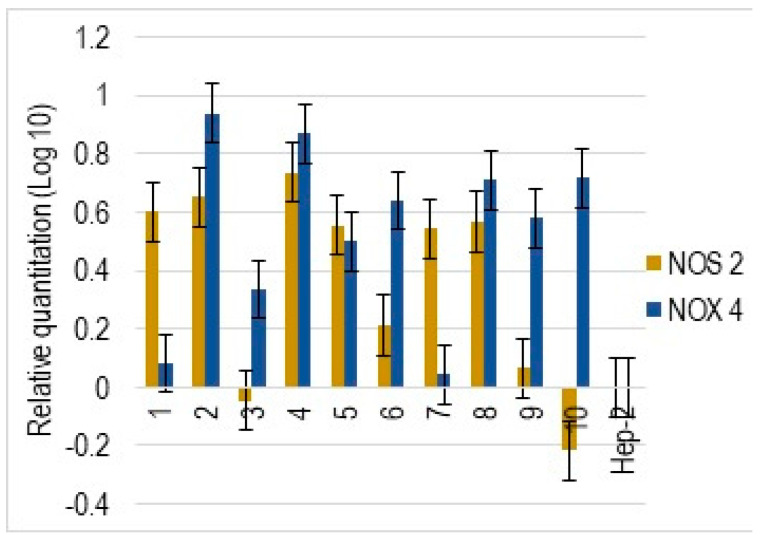
The effects of azulene-containing chalcones on NOS2 and NOX4 expression.

One of our approaches consisted of the analysis of NOS2 and NOX4 gene expression (Figure 6). The nitric oxide synthase (NOS2) is responsible for high-level NO production, and its activation is transcriptionally regulated by cytokines [45] that probably are produced during treatments. Azulene-containing chalcones induced NOS2 expression, with the exception of **3** and **10.** Moreover, in the case of **9**, the expression of NOS2 was reduced. It seems that the amino substitution might not be stable in the presence of NOS due to possible degradation by oxygen species. In addition, methyl substitution of the azulene moiety, located on either ring A or ring B of azulene, causes a decrease in the NOS2 expression. NADPH oxidase 4 (NOX4) is an enzyme that produces reactive oxygen species (ROS) by transferring electrons from NADPH to molecular oxygen.

All azulene-containing chalcones were involved in NOX4 activation, but the increase in the cases of **1** and **7** was minimal. All these results sustain the idea that azulene promotes intrinsic apoptosis through **NOX4-** and NOS2-mediated activation (Figure 6).

Quantitative structure−activity relationship (QSAR) studies involving 38 chalcone derivatives (Table 7) with known antibacterial activity against *S. aureus* [29,30,31,32,33] were developed. These studies were based upon observed activity and molecular descriptors. The experimental activity, A_exp_, was defined as follows: A_exp_ = 0.1 + log (*k*/MIC), with *k* = 1.250 for obtaining positive values of the activity (Table 7). Two QSAR studies were performed: (i) one in which all 48 molecules were included in the calibration set and (ii) another with an external validation test. For the latter, 10 molecules were randomly chosen to be part of the validation test, whereas the rest were part of the calibration set. The result of the external validation depends strongly, among other things, on the procedures for the extraction of the calibration and validation sets from the initial database [46,47,48].

To minimize the subjectivity in choosing the validation set, we included in this set the molecules having the ranks 1, 4, 9, 11, 17, 21, 24, 29, 32, and 37 and azulene-chalcones **2** and **10**. The other 36 molecules shown in Table 7 were used as the calibration set for building QSARs.

The calculated QSARs are multilinear equations:

$$A_{calc=C_{0\;}+\overset{p}{\underset{i=1}{\sum }}C_{i}\cdot D_{i}}$$

where *A_calc_* is the calculated value of the activity, *C*_0_ is the intercept, *C_i_* are weighting factors (coefficients), *D_i_* are (the values of some) descriptors, and *p* is the number of descriptors.

Taking *N* as the number of molecules in the calibration set, the agreement between the experimental and the calculated values (i.e., the quality of the QSARs) was measured using the usual statistical functions “square of linear Pearson correlation” *r*^2^, “Fisher function” *F*, and “standard error” SE. The value of *r*^2^ is in the 0–1 range. The values of *F* and SE are in the 0–∞ range.

*r*^2^ = A^2^/(B · C)

where A = Σ [(X_i_ − X_m_) ∙ (Y_i_ − Y_m_)]; B = Σ [(X_i_ − X_m_)^2^]; C = Σ [(Y_i_ − Y_m_)^2^]; X_i_ and Y_i_ are values of two certain variables; and X_m_ and Y_m_ are the average of X_i_ and Y_i_ values, respectively.


$$SE={\left[\frac{\sum {\left(A_{exp}-A_{calc}\right)}^{2}}{N-1}\right]}^{1/2}$$

In the first QSAR study, the best equation regarding the r^2^ value was C_0_ = −3.1308; C_1_ = 0.1219; D_1_ is number of (X,X) pairs (X is any halogen atom); C_2_ = 61.4760; D_2_ is the d_1_·d_2_ product (d_1,2_ are sums of repulsion forces on probe atoms #4 and #72 (3D descriptors)); C_3_ = −1.3011; D_3_ is the d_3_·d_4_ product (d_3_ is maximum parallax for probe atom #68 (3D descriptor), d_4_ is sum of repulsion forces on probe atom #37 (3D descriptor)); C_4_ = −0.2062; D_4_ is the d_5_·d_6_ product (d_5_ is minimum force in C-H bonds, d_6_ is 100·Max. nucleophilic reaction index for O atoms); C_5_ = 70.5984; D_5_ is average valence connectivity index χ^−4^ [49]; C_6_ = −1.5630; D_6_ is Moran autocorrelation #5, weighted by atomic vdW volumes [50].

The quality of the prediction is *p* = 6, N = 46, r^2^ = 0.9102, SE = 0.1808, and F = 67.5, with no outlier molecules. The calculated values of activity using this equation are listed in Table 7. A good correlation can be observed between experimental and predicted activity for the 48 compounds using this model. It can be said that there is a complex synergistic influence of the chemical structure, molecular shape, and molecular size on the activity. A high percentage (in weight) of halogens increases the value of activity, as does high molecular mass. The presence of halogen, or even CF_3_ groups, grafted in the 3 position and/or 5 position on the benzene ring, increases the activity. The presence of some predictors as products emphasizes certain synergistic effects of 3D positions of atomic charges.

In the second QSAR study, the best equation obtained with only 36 molecules is C_1_ = 0.0251; D_1_ is sum of distances (Å) in (X,Y) pairs, where X is any halogen atom and Y is any O atom linked to H atom; C_2_ = 1.5341, D_2_ is autocorrelation #6, weighted by Sanderson electronegativities [51]; C_3_ = 3.9608, D_3_ is the d_1_·d_2_ product (d_1_ is maximum parallax for probe atom #43 (3D descriptor), d_2_ is sum of repulsion forces on probe atom #28 (3D descriptor)); C_4_ = 57.2208, D_4_ is average valence connectivity index χ^−4^; C_5_ = −9.5190, D_5_ is topological charge index order 10 [52].

The quality of the prediction for the molecules used in the calibration set is *p* = 6, N = 66, r^2^ = 0.8903, SE = 0.2100, and F = 50.3, and the quality of the validation set is r^2^ = 0.9034 and SE = 0.1984. Surprisingly, the quality of the prediction for the validation set is a bit higher than the quality of the prediction for the calibration set. The quality of the prediction for calibration and validation sets can be considered “good” and “comparable”. Therefore, both the calibration set and the validation set seem to be “representative sample” for the molecules listed in Table 7.

## 4. Conclusions

We described the synthesis of 10 novel azulene-chalcone derivatives and investigated whether azulene moieties enhance the biological properties of classical chalcone derivatives. Their effectiveness as antibacterial drugs was investigated against Gram-positive and Gram-negative bacteria. All compounds showed moderate activity against Gram-negative bacteria and low antimicrobial activity against *S. aureus*. The prepared compounds displayed antifungal activity against *C. parapsilosis* strain with higher efficiency induced by azulene moieties.

Structurally speaking, the substitution of one or both phenyl rings of the parent chalcone compound with azulene group(s) resulted in the redshift of the long-wavelength absorption band over 420 nm. The synthesized azulene-containing chalcones showed fluorescent properties in the 450–700 nm range, depending on the number of azulene fragments and the substituent position in the azulene fragment and position relative to the ketone group. Moreover, quantitative structure–activity relationship (QSAR) studies for antibacterial activity against *S. aureus* of reported and herein-described chalcones were developed. A good correlation was observed between experimental and predicted activity for the 48 compounds using the models involved in the prediction of the antibacterial activity. The quality of the prediction was influenced by the substitution of the chalcone structures; thus, two approaches were used for obtaining an accurate prediction.

## Figures and Tables

**Table 1:** Substitution patterns in Type I chalcones and the reaction yield.

Compound	Ar	Yield (%)
**1**	Phenyl	67
**2**	4-OH-Phenyl	73
**3**	4-NH_2_-Phenyl	62
**4**	1-Naphthyl	75
**5**	2-Naphthyl	95

**Table 2:** Substitution patterns in Type II chalcones and the reaction yield.

Compound	R	R′	Yield (%)
**6**	H	H	84
**7**	H	Cl	78
**8**	H	Br	74
**9**	H	CH_3_	63
**10**	CH_3_	H	57

**Table 3:** Relevant ^1^H-chemical shifts for azulene-containing chalcones.

Compd.	H-α	H-β	Az-2	Az-3	Az-4	Az-8
**1**	7.61	8.54	8.35	7.46	8.34	8.69
**2**	7.90	8.37	8.65	7.55	8.48	8.80
**3**	7.63	8.50	8.34	7.45	8.32	8.79
**4**	7.37	8.32	8.30	7.44	8.31	8.50
**5**	7.78	8.60	8.43	7.48	8.36	8.72
**6**	7.83	8.55	8.40	7.37	8.32	8.74
**7**	7.82	8.64	8.28	-	8.39	8.68
**8**	7.79	8.46	8.35	-	8.36	8.68
**9**	7.79	8.51	8.22	-	8.19	8.63
**10**	7.79	8.52	8.39	7.45	8.29	8.71

**Table 4:** UV-Vis absorption and emission bands of azulene-containing chalcones.

Compd.	λ_nm_ (log ε)/Band I	λ_nm_ (log ε)/Band II	λ_em_, nm
**1**	425 (4.52)	326 (4.14), 283 (4.35)	542(bb *)/475(sh **)/**484**/521(bb)
**2**	423 (4.42)	320 (4.14), 293 (4.21)	451 (bb)/474(sh)/**485**/525 (bb)
**3**	421 (4.54)	339 (4.24), 280 (4.50), 262 (4.54)	452(bb)/472(sh)/**484**/525(bb)/650(bb)
**4**	423 (4.46)	328 (4.20), 288 (4.29)	450 (bb)/471(sh)/**484**/525(bb)
**5**	431 (4.50)	334 (4.11), 289 (4.45), 262 (4.47)	451(bb)/474(sh)/**485**/525(bb)
**6**	451 (4.59), 436 (4.58)	322 (4.33), 288 (4.47)	452(bb)/473(sh)/**485**/525(bb)
**7**	453 (4.71), 438 (4.04)	322 (3.92), 291 (4.02)	452(bb)/474(sh)/**484**/525(bb)
**8**	453 (4.71), 439 (4.70)	324 (4.47), 290 (4.58)	452(bb)/473(sh)/**485**/525(bb)
**9**	461 (4.32), 447 (4.31)	323 (4.06), 291 (4.24)	451(bb)/471(sh)/**485**/525(bb)
**10**	462 (4.62)	330 (4.38), 291 (4.59)	452(bb)/**486**/525/566(bb)/**654**

**Note**: * broad band; ** shoulder; the maximum emission bands are bold written.

**Table 5:** Antibacterial and antifungal activities of azulene-containing chalcones **1**–**10**.

Microorganisms	Compounds (MIC mg/mL)									
	1	2	3	4	5	6	7	8	9	10
*Staphylococcus aureus*	1.250	1.250	1.250	1.250	1.250	0.625	0.625	1.250	1.250	1.250
*Escherichia coli*	0.625	0.625	0.625	0.625	0.625	0.625	0.625	0.625	0.625	0.625
*Pseudomonas aeruginosa*	0.625	0.625	0.625	0.625	0.625	0.625	0.625	0.625	0.625	0.625
*Candida parapsilosis*	0.312	0.312	0.312	0.312	0.312	0.156	0.312	0.312	0.312	0.312

**Table 6:** Antibiofilm activity of azulene-containing chalcones **1**–**10**.

Microorganisms	Compounds (MBEC mg/mL)									
	1	2	3	4	5	6	7	8	9	10
*Staphylococcus aureus*	1.250	1.250	2.500	1.250	2.500	0.625	0.625	1.250	0.625	1.250
*Escherichia coli*	0.625	0.625	0.625	0.625	0.625	0.625	1.250	0.625	0.625	0.625
*Pseudomonas aeruginosa*	0.625	0.625	1.250	0.625	1.250	0.625	0.625	1.250	0.625	0.625
*Candida parapsilosis*	0.312	0.312	0.625	0.625	0.625	0.156	0.312	0.312	0.312	0.312

**Table 7:** Antibacterial activity against *S. aureus* of the chalcones used for QSAR studies.

Nr. Crtl.		R_1_	R_2_	MIC (mg/mL)	A_exp_	QSAR Study I	QSAR Study II
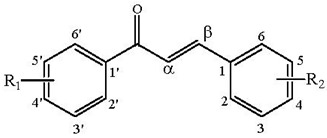							
1		H	4-OH, 3-OCH_3_	0.500	0.498	0.416	0.698
2		4′-Cl	4-OH, 3,5-diOCH_3_	0.500	0.498	0.460	0.556
3		4′-OH-3′-F	2,4-diCl	0.300	0.720	1.234	1.386
4		4′-COOH	2-CF_3_	0.300	0.720	0.534	1.013
5		4′-COOH	3-NO_2_	0.300	0.720	1.314	0.748
6		H	3-OH	0.250	0.799	0.987	0.604
7		H	3-OH, 4-OCH_3_	0.250	0.799	1.015	0.684
8		4′-Cl	2-OH, 3-OCH_3_	0.250	0.799	0.746	1.085
9		4′-Cl	4-OH, 3-OCH_3_	0.250	0.799	0.646	1.123
10		4′-OH-3′,5′-diF	2,4-diCl	0.150	1.021	1.111	0.925
11		4′-COOH	4-CF_3_	0.150	1.021	0.837	2.049
12		4′-COOH	4-Cl	0.150	1.021	0.998	1.496
13		4′-COOH	2-Cl	0.150	1.021	0.961	0.953
14		4′-COOH	2-Br	0.150	1.021	1.010	1.112
15		4′-COOH	3,5-diF	0.150	1.021	0.931	1.323
16		H	4-OH	0.125	1.100	1.234	1.090
17		H	3,4-diOH	0.125	1.100	0.638	0.651
18		H	2-OH, 3-OCH_3_	0.125	1.100	0.670	0.738
19		4′-Cl	2-OH	0.125	1.100	1.238	1.279
20		4′-Cl	3-OH	0.125	1.100	0.866	0.866
21		4′-Cl	4-OH	0.125	1.100	1.136	1.475
22		4′-Cl	3,4-diOH	0.125	1.100	0.805	1.110
23		4′-COOH	3-Cl	0.075	1.322	1.296	1.223
24		4′-COOH	3-Br	0.075	1.322	1.449	1.664
25		4′-COOH	3,5-diCH_3_	0.075	1.322	1.289	1.322
26		4′-Cl	3-OH, 4-OCH_3_	0.0625	1.401	1.200	1.156
27		H	2-OH	0.0625	1.401	1.420	0.918
28		4′-COOH	3-CF_3_	0.040	1.595	1.243	1.549
29		4′-COOH	3,5-diCl	0.040	1.595	1.547	1.764
30		4′-COOH	2,4-diCl	0.040	1.595	1.648	1.492
31		4′-OH	2,4-diCl	0.020	1.896	1.649	1.953
32		4′-OH-2-F	2,4-diCl	0.020	1.896	1.734	1.165
33		4′-OH	3-Br	0.020	1.896	1.403	2.034
34		4′-OH	2,3-diCl	0.015	2.021	1.651	1.890
35		4′-OH	2,3,6-triCl	0.010	2.197	1.829	2.059
36		4′-OH	3,5-diCF_3_	0.003	2.720	2.399	2.660
37		4′-COOH	3,5-diCF_3_	0.002	2.896	2.521	2.547
38		4′-COOH	3,5-diBr	0.002	2.896	2.227	2.664
	Compounds synthesized herein						
39	**1**			1.250	0.100	0.233	0.244
40	**2**			1.250	0.100	0.023	1.395
41	**3**			1.250	0.100	0.213	0.233
42	**4**			1.250	0.100	0.119	0.245
43	**5**			1.250	0.100	0.044	0.246
44	**6**			0.625	0.401	−0.027	0.183
45	**7**			0.625	0.401	0.488	0.223
46	**8**			1.250	0.100	0.492	0.175
47	**9**			1.250	0.100	−0.069	0.184
48	**10**			1.250	0.100	−0.068	0.172

## Data Availability

Samples of the compounds are not available from the authors.

## Supplementary Materials

The following supporting information can be downloaded at: https://www.mdpi.com/article/10.3390/ma15051629/s1. Figure S1: Overlay of the UV-Vis spectra of azulene-chalcone derivatives, 1–10 recorded in dichloromethane; Figure S2: Azulene effects on the cell cycle for HEp2. Histograms show the changes in cellular DNA content after treatment with 50 µg/mL azulene.

## References

[1] Zhuang C., Zhang W., Sheng C., Zhang W., Xing C., Miao Z. (2017). Chalcone: A Privileged Structure in Medicinal Chemistry. Chem. Rev..

[2] Thapa P., Upadhyay S.P., Suo W.Z., Singh V., Gurung P., Lee E.S., Sharma R., Sharma M. (2021). Chalcone and its analogs: Therapeutic and diagnostic applications in Alzheimer’s disease. Bioorg. Chem..

[3] Turkovic N., Ivkovic B., Kotur-Stevuljevic J., Tasic M., Marković B., Vujic Z. (2020). Molecular Docking, Synthesis and anti-HIV-1 Protease Activity of Novel Chalcones. Curr. Pharm. Des..

[4] Upadhyay N., Tilekar K., Loiodice F., Anisimova N.Y., Spirina T.S., Sokolova D.V., Smirnova G.B., Choe J.-Y., Meyer-Almes F.-J., Pokrovsky V.S. (2021). Pharmacophore hybridization approach to discover novel pyrazoline-based hydantoin analogs with anti-tumor efficacy. Bioorg. Chem..

[5] Ismail M.M., Farrag A.M., Harras M., Ibrahim M.H., Mehany A.B. (2020). Apoptosis: A target for anticancer therapy with novel cyanopyridines. Bioorganic Chem..

[6] Phan C.-W., Sabaratnam V., Yong W.-K., Malek S.N.A. (2017). The role of chalcones: Helichrysetin, xanthohumol, and flavokawin-C in promoting neurite outgrowth in PC12 Adh cells. Nat. Prod. Res..

[7] Dhar D.N. (2003). The Chemistry of Chalcones and Related Compounds.

[8] Ngaini Z., Fadzillah S.M.H., Hussain H. (2012). Synthesis and antimicrobial studies of hydroxylated chalcone derivatives with variable chain length. Nat. Prod. Res..

[9] Rocha J.E., de Freitas T.S., Xavier J.D.C., Pereira R.L.S., Junior F.N.P., Nogueira C.E.S., Marinho M.M., Bandeira P.N., de Oliveira M.R., Marinho E.S. (2021). Antibacterial and antibiotic modifying activity, ADMET study and molecular docking of synthetic chalcone (E)-1-(2-hydroxyphenyl)-3-(2,4-dimethoxy-3-methylphenyl)prop-2-en-1-one in strains of Staphylococcus aureus carrying NorA and MepA efflux pumps. Biomed. Pharmacother..

[10] Ávila H.P., Smânia E.D.F.A., Monache F.D., Smânia A. (2008). Structure–activity relationship of antibacterial chalcones. Bioorg. Med. Chem..

[11] El-Meligie S., Taher A.T., Kamal A.M., Youssef A. (2017). Design, synthesis and cytotoxic activity of certain novel chalcone analogous compounds. Eur. J. Med. Chem..

[12] Wachter N.M., Rani N., Zolfaghari A., Tarbox H., Mazumder S. (2020). DFT investigations of the unusual reactivity of 2-pyridinecarboxaldehyde in base-catalyzed aldol reactions with acetophenone. J. Phys. Org. Chem..

[13] Zeller K.P., Kropf H. (1985). Azulene, in Methoden der Organischen Chemie (Houben-Weyl).

[14] Dragu E.A., Ion A.E., Shova S., Bala D., Mihailciuc C., Voicescu M., Ionescu S., Nica S. (2015). Visible-light triggered photoswitching systems based on fluorescent azulenyl-substituted dithienylcyclopentenes. RSC Adv..

[15] Yamaguchi Y., Ogawa K., Nakayama K.-I., Ohba Y., Katagiri H. (2013). Terazulene: A High-Performance n-Type Organic Field-Effect Transistor Based on Molecular Orbital Distribution Control. J. Am. Chem. Soc..

[16] Murai M., Iba S., Ota H., Takai K. (2017). Azulene-Fused Linear Polycyclic Aromatic Hydrocarbons with Small Bandgap, High Stability, and Reversible Stimuli Responsiveness. Org. Lett..

[17] Ion A.E., Nica S., Madalan A.M., Maxim C., Julve M., Lloret F., Andruh M. (2014). One-dimensional coordination polymers constructed from di- and trinuclear {3d–4f} tectons. A new useful spacer in crystal engineering: 1,3-bis(4-pyridyl)azulene. CrystEngComm.

[18] Ion A.E., Dogaru A., Shova S., Madalan A.M., Akintola O., Ionescu S., Voicescu M., Nica S., Buchholz A., Plass W. (2018). Organic co-crystals of 1,3-bis(4-pyridyl)azulene with a series of hydrogen-bond donors. CrystEngComm.

[19] Dong J.-X., Zhang H.-L. (2016). Azulene-based organic functional molecules for optoelectronics. Chin. Chem. Lett..

[20] Razus A.C., Nica S., Cristian L., Raicopol M., Birzan L., Dragu A.E. (2011). Synthesis and physico-chemical properties of highly conjugated azo-aromatic systems. 4-(azulen-1-yl)-pyridines with mono- and bis azo-aromatic moieties at C3-position of azulene. Dye. Pigment..

[21] Srivastava J., Pandey M., Gupta S. (2009). Chamomile, a novel and selective COX-2 inhibitor with anti-inflammatory activity. Life Sci..

[22] Löber S., Tschammer N., Hübner H., Melis M.R., Argiolas A., Gmeiner P. (2009). The Azulene Framework as a Novel Arene Bioisostere: Design of Potent Dopamine D4 Receptor Ligands Inducing Penile Erection. ChemMedChem.

[23] Akagi M., Matsui N., Mochizuki S., Tasaka K. (2001). Inhibitory effect of egualen sodium: A new stable derivative of azulene on histamine release from mast cell-like cells in the stomach. Pharmacology.

[24] Dragu E.A., Nica S., Tecuceanu V., Bala D., Mihailciuc C., Hanganu A., Razus A.C. (2013). Synthesis and Electrochemical Properties of Carbocyclic and Heterocyclic Diazulenylethenes. Eur. J. Org. Chem..

[25] Razus A.C., Pavel C., Lehadus O., Nica S., Birzan L. (2008). Synthesis and properties of [1,6′]biazulenyl compounds. Tetrahedron.

[26] Dragu E.A., Nica S., Raicopol M., Baran A., Anghel D.-F., Cojocaru B., Tarko L., Razus A.C. (2012). Synthesis, solid-state photophysical properties and electropolymerization of novel diazulenyl ethenes. Tetrahedron Lett..

[27] Ion A.E., Cristian L., Voicescu M., Bangesh M., Madalan A.M., Bala D., Mihailciuc C., Nica S. (2016). Synthesis and properties of fluorescent 4′-azulenyl-functionalized 2,2′:6′,2″-terpyridines. Beilstein J. Org. Chem..

[28] Cinteza L.O., Voicu S.N., Popa M., Marutescu L., Nitu S., Somoghi R., Nistor C.L., Petcu C. (2018). Rational design of silver nanoparticles with reduced toxicity and enhanced antimicrobial activity. Rom. Biotechnol. Lett..

[29] (2018). Antifungal activity of some medicinal plant extracts against Candida albicans nosocomial isolates. Rom Biotechnol Lett.

[30] Nuţă D.C., Măruţescu L., Missir A.V., Morușciag L., Chiriţă C., Curuţiu C., Bădiceanu C.D., Papadocea M.T., Limban C. (2017). In vitro evaluation of the antimicrobial activity of N-phenylcarbamothioyl benzamides against planktonic and adherent microbial cells. Rom. Biotechnol. Lett..

[31] Nielsen S.F., Larsen M., Boesen T., Schønning K., Kromann H. (2005). Cationic Chalcone Antibiotics. Design, Synthesis, and Mechanism of Action. J. Med. Chem..

[32] Katsori A.-M. (2009). Chalcones in Cancer: Understanding their Role in Terms of QSAR. Curr. Med. Chem..

[33] Nielsen S.F., Boesen T., Larsen M., Schønning K., Kromann H. (2004). Antibacterial chalcones––bioisosteric replacement of the 4′-hydroxy group. Bioorg. Med. Chem..

[34] Batovska D.I., Todorova D.I.B.A.I.T. (2010). Trends in Utilization of the Pharmacological Potential of Chalcones. Curr. Clin. Pharmacol..

[35] Batovska D., Parushev S., Stamboliyska B., Tsvetkova I., Ninova M., Najdenski H. (2009). Examination of growth inhibitory properties of synthetic chalcones for which antibacterial activity was predicted. Eur. J. Med. Chem..

[36] Gajewski J.J., Gilbert K.E. (1992). PCMODEL Molecular Modeling Package.

[37] Laszlo T. (2017). A selection method for molecular descriptors and QSPR equations. MATCH Commun. Math Comput Chem..

[38] Nielsen A.T., Smith M.B., March J. (2001). Advanced Organic Chemistry.

[39] Hergert H.L., Kurth E.F. (1953). The Infrared Spectra of Lignin and Related Compounds. I. Characteristic Carbonyl and Hydroxyl Frequencies of Some Flavanones, Flavones, Chalcones and Acetophenones. J. Am. Chem. Soc..

[40] Pelter A., Ward R.S., Gray T.I. (1976). The carbon-13 nuclear magnetic resonance spectra of flavonoids and related compounds. J. Chem. Soc. Perkin Trans. 1.

[41] Black W.B., Lutz R.E. (1955). Ultraviolet Absorption Spectra of Chalcones. Identification of Chromophores. J. Am. Chem. Soc..

[42] Xue Y., Mou J., Liu Y., Gong X., Yang Y., An L. (2010). An ab initio simulation of the UV/Visible spectra of substituted chalcones. Open Chem..

[43] Emiquel S., Elagrafeuille R., Esouweine B., Eforestier C. (2016). Anti-biofilm Activity as a Health Issue. Front. Microbiol..

[44] Boland K., Flanagan L., Prehn J.H. (2013). Paracrine control of tissue regeneration and cell proliferation by Caspase-3. Cell Death Dis..

[45] De Vera M.E., Shapiro R.A., Nussler A.K., Mudgett J.S., Simmons R.L., Morris S.M., Billiar T.R., Geller D.A. (1996). Transcriptional regulation of human inducible nitric oxide synthase (NOS2) gene by cytokines: Initial analysis of the human NOS2 promoter. Proc. Natl. Acad. Sci. USA.

[46] Draper N.R., Smith H. (1981). Applied Regression Analysis.

[47] Leonard J.T., Roy K. (2006). On Selection of Training and Test Sets for the Development of Predictive QSAR models. QSAR Comb. Sci..

[48] Xu G., Hughes-Oliver J., Brooks J., Yeatts J., Baynes R. (2013). Selection of appropriate training and validation set chemicals for modelling dermal permeability by U-optimal design. SAR QSAR Environ. Res..

[49] Kier L.B., Hall L.H. (1981). Derivation and Significance of Valence Molecular Connectivity. J. Pharm. Sci..

[50] Moran P.A.P. (1950). Notes on continuous stochastic phenomena. Biometrika.

[51] Consonni V., Todeschini R., Pavan M., Gramatica P. (2002). Structure/Response Correlations and Similarity/Diversity Analysis by GETAWAY Descriptors. 2. Application of the Novel 3D Molecular Descriptors to QSAR/QSPR Studies. J. Chem. Inf. Comput. Sci..

[52] Galvez J., Garcia R., Salabert M.T., Soler R. (1994). Charge Indexes. New Topological Descriptors. J. Chem. Inf. Comput. Sci..

