# Supplementary material for: Design, Synthesis, and Biological Evaluation of New Azulene-Containing Chalcones
Source: Materials (Basel). 2022 Feb 22;15(5):1629. doi: 10.3390/ma15051629 (PMC8911025; doi:10.3390/ma15051629)
Supplement: Supplementary file 1 [file materials-15-01629-s001.zip › materials-1544486-supplementary.pdf]

Design, Synthesis, and Biological Evaluation of New Azulene-Containing Chalcones

Daniela Bala [1], Luiza-Izabela Jinga [2], Marcela Popa [3,4], Anamaria Hanganu [2,5], Mariana Voicescu [6], Coralia Bleotu [3,4,7], Laszlo Tarko [2] and Simona Nica [2,*]

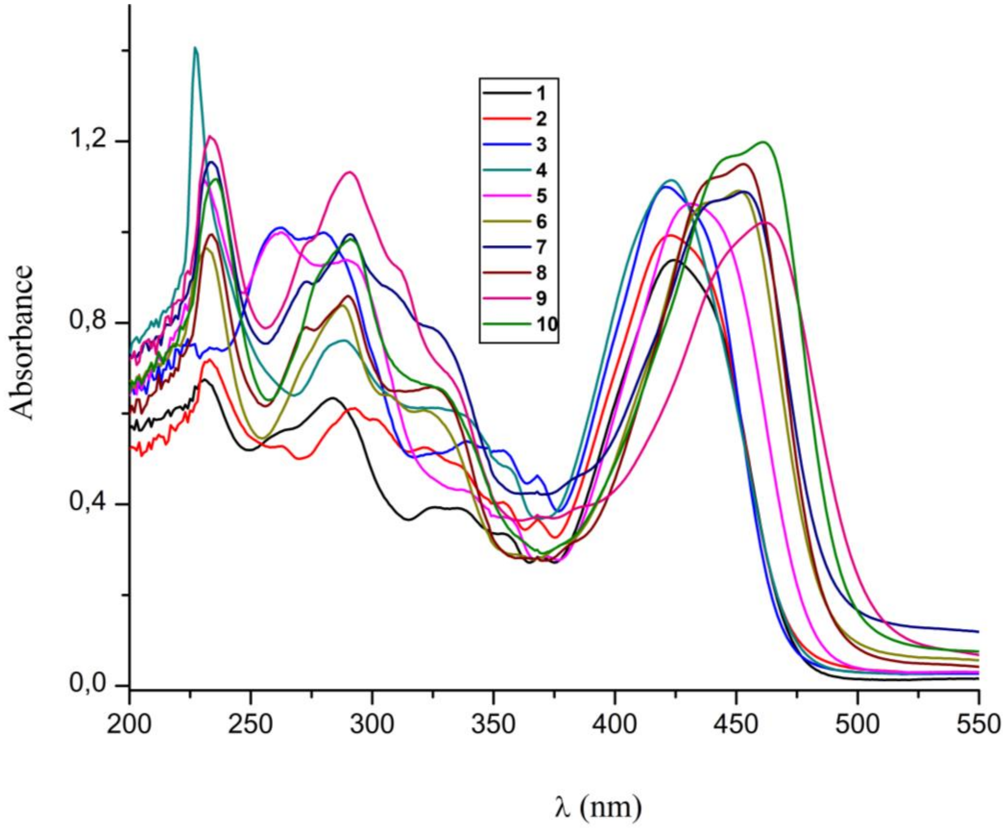

Figure S1. Overlay of the UV-Vis spectra of azulene-chalcone derivatives, **1 – 10 recorded** in dichloromethane.

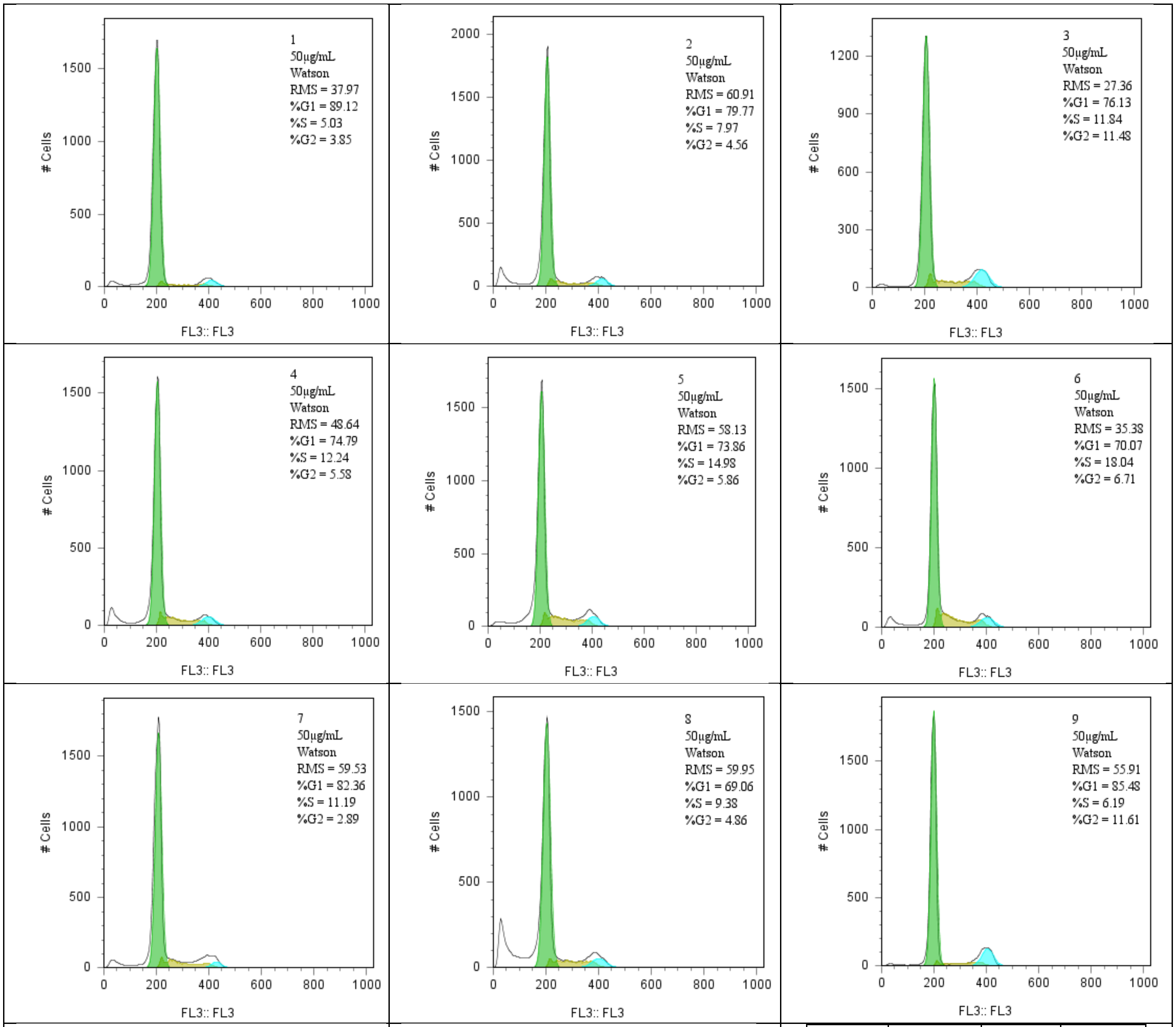

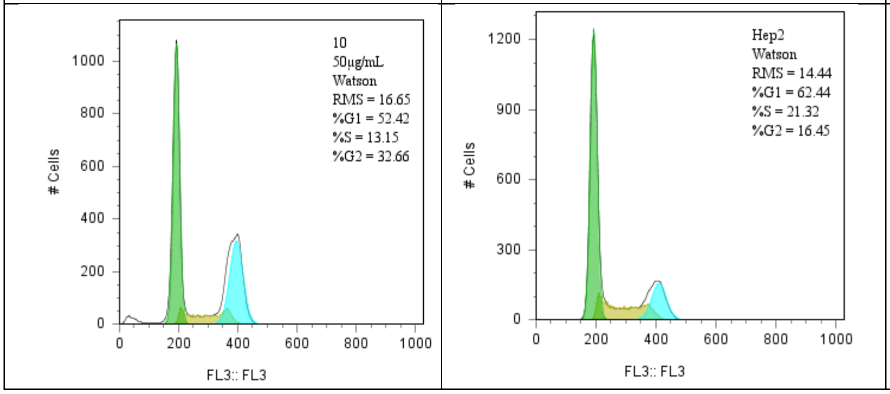

	G0/G1	S	G2/M
1	89.12	5.03	3.85
2	79.77	7.97	4.56
3	76.13	11.84	11.48
4	74.79	12.24	5.58
5	73.86	14.98	5.86
6	70.07	18.04	6.71
7	82.36	11.19	2.89
8	69.06	9.38	4.86
9	85.48	6.19	11.61
10	52.42	13.15	32.66
HEep2	62.44	21.32	16.45

Figure S2. Azulene effects on the cell cycle for HEp2. Histograms show the changes in cellular DNA content after treatment with 50µg/mL azulene.
